# Latently KSHV-Infected Cells Promote Further Establishment of Latency upon Superinfection with KSHV

**DOI:** 10.3390/ijms222111994

**Published:** 2021-11-05

**Authors:** Chen Gam ze Letova, Inna Kalt, Meir Shamay, Ronit Sarid

**Affiliations:** 1 The Mina and Everard Goodman Faculty of Life Sciences, Bar-Ilan University, Ramat Gan 5290002, Israel; gamzolc@gmail.com (C.G.z.L.); Inna.Kalt@biu.ac.il (I.K.); 2 Advanced Materials and Nanotechnology Institute, Bar-Ilan University, Ramat Gan 5290002, Israel; 3 Daniella Lee Casper Laboratory in Viral Oncology, Azrieli Faculty of Medicine, Bar-Ilan University, Safed 1311502, Israel; meir.shamay@biu.ac.il

**Keywords:** Kaposi’s sarcoma-associated herpesvirus (KSHV), superinfection, primary infection, latency-associated nuclear antigen 1 (LANA-1), latent infection

## Abstract

Kaposi’s sarcoma-associated herpesvirus (KSHV) is a cancer-related virus which engages in two forms of infection: latent and lytic. Latent infection allows the virus to establish long-term persistent infection, whereas the lytic cycle is needed for the maintenance of the viral reservoir and for virus spread. By using recombinant KSHV viruses encoding mNeonGreen and mCherry fluorescent proteins, we show that various cell types that are latently-infected with KSHV can be superinfected, and that the new incoming viruses establish latent infection. Moreover, we show that latency establishment is enhanced in superinfected cells compared to primary infected ones. Further analysis revealed that cells that ectopically express the major latency protein of KSHV, LANA-1, prior to and during infection exhibit enhanced establishment of latency, but not cells expressing LANA-1 fragments. This observation supports the notion that the expression level of LANA-1 following infection determines the efficiency of latency establishment and avoids loss of viral genomes. These findings imply that a host can be infected with more than a single viral genome and that superinfection may support the maintenance of long-term latency.

## 1. Introduction

Kaposi’s sarcoma-associated herpesvirus (KSHV), also referred to as human herpesvirus-8 (HHV-8), is a gamma-2 herpesvirus that is implicated in the etiology of Kaposi’s sarcoma (KS), primary effusion lymphoma (PEL), plasmablastic variant of multicentric Castleman’s disease and KSHV-inflammatory cytokine syndrome (KICS) [1,2,3,4,5,6,7]. Like all herpesviruses, primary infection with KSHV results in life-long infection, which is mostly latent, coupled with sporadic lytic reactivation episodes that produce new virions and enable virus dissemination between hosts and within the host, while increasing the risk for disease development [8,9]. This biphasic infection cycle has been widely explored using various cell models in which a reversible latent infection can be shifted by different physiological and chemical treatments toward the productive/lytic infection phase [9,10]. Of note, KSHV appears to display an unstable latency with a propensity to lose latent viral genomes; thus, it is likely that lytic reactivation is required to produce newly infected cells within the host, and could be vital for sustaining long-term host infection [8,11,12]. 

Latency of KSHV is characterized by persistence of multiple highly ordered covalently closed circular viral genomes, termed episomes, that assemble into chromatin. A limited set of viral genes are expressed during latency, while the viral DNA replicates using the host DNA replication machinery coordinately with host cell division. Establishment of KSHV latency within a cell involves spatially and temporally ordered chromatinization, including a rapid association with histones that are enriched with H3K4me3 and H3K27ac activating marks, and concomitant expression of viral lytic genes, followed by a transition to H3K27me3/H2AK119ub-enriched heterochromatin, which ultimately results in the repression of viral gene expression [13,14]. Enrichment of repressive chromatin marks were also detected in KS tissues and in latently infected PEL cells [15]. The epigenetic modifications are predominantly orchestrated by the viral latency-associated nuclear antigen 1 (LANA-1). LANA-1 is a multifunctional DNA-binding protein, which is expressed during KSHV latency and known to be vital for various nuclear functions, in particular for the preservation of KSHV episomes and the segregation of the replicated viral genomes into daughter cells during latency. LANA-1 performs these functions through the recruitment of cellular proteins, including replication factors, chromatin modifying enzymes, and cellular mitotic apparatus assembly [16,17]. 

The process by which already infected cells are re-infected with another virus of a different or similar type is called superinfection. In this context, a phenomenon known as superinfection exclusion, characterized by the inhibition of subsequent viral infection by an infected cell, has been described [18]. This phenomenon, suggested to preserve the cell’s resources toward promotion of replication and spread of the virus that initially infected the cell, was first observed for bacteriophages [19] and later in a variety of viruses including influenza virus [20], poxviruses [21], flaviviruses [22,23,24], alphaviruses [25], and papillomaviruses [26]. Inhibition of the second infecting virus was also reported in three herpesviruses, and herpes simplex virus type 1 (HSV-1) [27,28], varicella-zoster virus (VZV), and porcine herpesvirus pseudorabies virus (PRV), displaying both self and mutual superinfection exclusion during sequential infection [29,30]. Resistance of trigeminal neurons to subsequent HSV-1 infection was reported in vivo [31]; yet, variations were reported in the viruses shed by individuals from herpetic lesions over time, presumably resulting from sequential infections [32]. Reinfection with VZV and subsequent latency establishment may also occur [33]. Of note, co-infection with different herpesvirus strains enables recombination-mediated diversification and serves as a driving force for the emergence of new strains [33,34,35,36,37,38]. Indeed, recent genome-wide sequence analyses provided evidence for intragenic and multiple recombination events contributing to the divergence of KSHV [39]. However, in which host cells, and at what stage co-infection occurs in infected hosts, is yet to be discovered.

Among human viruses, KSHV is most closely related to the Epstein-Barr virus (EBV), a gamma-1 herpesvirus associated with lymphomas and nasopharyngeal carcinoma. EBV and KSHV establish persistent infections in B cells and are found together in approximately 90% of PELs [40,41,42,43]. Increased EBV lytic gene expression and associated tumorigenesis are evident following co-infection with KSHV [44]. In addition, it has been observed that EBV promotes cell survival and KSHV persistence in dually infected cells [45,46], and that prolonged co-infection with these viruses leads to an increase in the expression of EBV lytic gene products, and at the same time enhances cancer cell transformation [44]. KS and PEL usually develop as a result of infection with a single genotype of KSHV; however, KS lesions carrying two different genotypes of KSHV have been identified [47,48,49]. In addition, a significant genotypic polymorphism within different compartments, blood, saliva, and KS lesions, was identified in KSHV-infected Malawian individuals [50]. Of note, recent whole-genome sequencing of KSHV-related effusions provided evidence not only for co-infection with EBV but also with the human herpesvirus 6 (HHV-6) and torque teno virus (TTV), suggesting that the effusions may contain multiple viral species [49]. This analysis also identified an effusion in an African-American patient which was co-infected by two different KSHV strains, the B or African subtype, and the A Europe or North America K1 subtype. However, whether both genomes were present in the same cells remains to be determined [49,51]. Similarly, infection with more than one EBV genotype has been described in healthy carriers of the virus, as well as in infectious mononucleosis and in AIDS patients [52,53,54].

In the present study, to increase our understanding of co-infection, we constructed recombinant viruses encoding mNeonGreen or mCherry fluorescent proteins and used these viruses to monitor primary infection of naive uninfected cells compared with corresponding superinfection of latently-infected cells. We show that latently KSHV-infected cells are permissive to secondary KSHV infection and that latency of the secondary incoming viruses is established more efficiently during superinfection compared to primary infection. Enhancement of latency establishment of KSHV infection was also evident upon expression of full-length LANA-1, suggesting that ongoing prior expression of LANA-1 supports latency establishment and prevents loss of viral genomes. These findings imply that a given host can be infected with more than a single viral genome and that superinfection may support the maintenance of long-term infection.

## 2. Results

### 2.1. Construction of BAC16-mCherry and BAC16-mNeonGreen Recombinant Viruses

**Figure 1:**
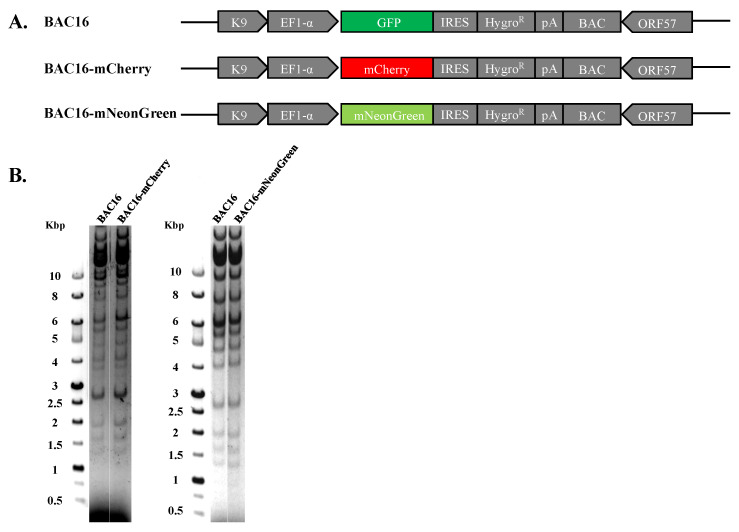
Construction and analysis of BAC16-mCherry and mNeonGreen KSHV recombinant genomes. (**A**) Schematic illustration of the parental KSHV BAC16 genome as well as BAC16-mCherry and BAC16-mNeonGreen genomes. (**B**) Agarose gel electrophoresis of BAC16, BAC16-mCherry, and BAC16-mNeonGreen. DNAs were digested with BglII, resolved on a 0.4% agarose gel, and stained with ethidium bromide. Molecular weight markers are shown on the left. BAC16-mCherry and BAC16-mNeonGreen display similar digestion pattern as that obtained for the corresponding BAC16 DNA.

Recombinant viral genomes that express mCherry or mNeonGreen fluorescent protein under the control of the constitutive cellular promoter EF1-alpha were constructed using the complete KSHV BAC16 clone, which enables genetic manipulation of the KSHV genome in *E. coli* employing the two-step Red-mediated recombination approach [55,56]. The resulting genomes, designated BAC16-mCherry or BAC16-mNeonGreen, were tested for the presence of the engineered exchange using diagnostic PCR, sequencing and restriction enzyme analysis with Bglll revealing DNA fragmentation pattern similar to that of parental BAC16 and lack of rearrangements (Figure 1). Of note, mCherry and mNeonGreen do not share sequence homology, thereby avoiding homologous recombination between the fluorescent genes during co-infection with both viruses. Furthermore, since the excitation and emission spectra of these proteins do not overlap, we anticipated that it would be possible to detect combined infections with both viruses.

### 2.2. Latently KSHV-Infected Cells Permit Superinfection and Display Enhanced Expression of the Fluorescent Marker of the Incoming Virions

**Figure 2:**
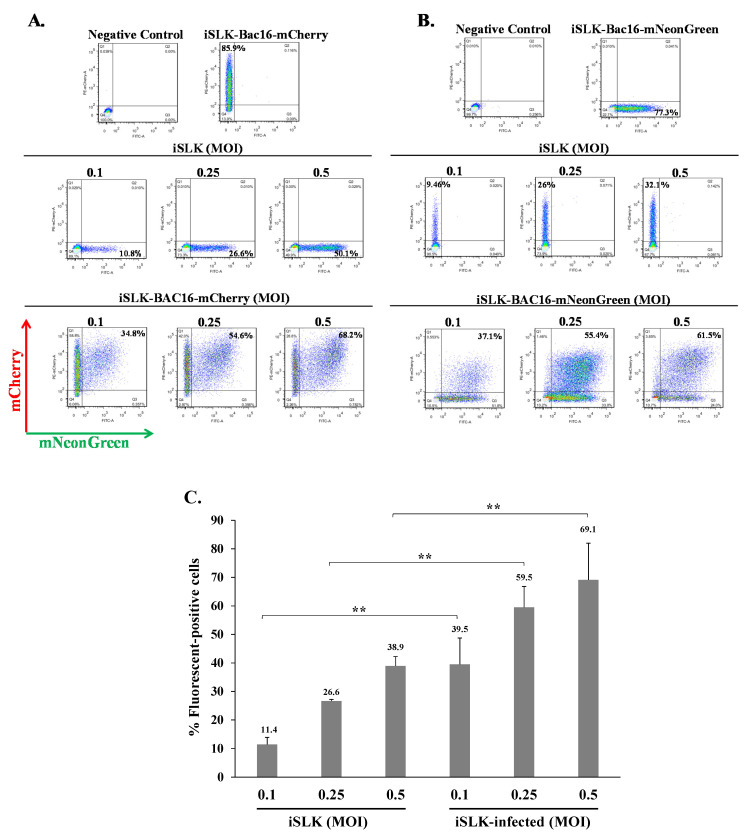
KSHV-infected iSLK cells can be superinfected and display enhanced expression of the fluorescent marker of the incoming virions. Naive iSLK cells and latently infected iSLK cells that carry (**A**) BAC16-mCherry or (**B**) BAC16-mNeonGreen were seeded in six-well plates (500,000 cells per well). The next day, the cells were infected with (**A**) BAC16-mNeonGreen or (**B**) BAC16-mCherry viruses at MOIs of 0.1, 0.25, and 0.5 using spinoculation. After 48 h, the cells were harvested, and the percentages of cells expressing mNeonGreen and mCherry were measured by FACS. (**C**) Percentage of cells expressing the fluorescent protein (mNeonGreen/mCherry) encoded by the incoming viral genome. iSLK—naive uninfected cells, iSLK-infected-latently iSLK-infected cells that were re-infected. Results show the average of three independent experiments. Statistical analysis was carried out using Tukey’s post hoc analysis test. ** *p* < 0.01. Naive uninfected iSLK cells were used as a negative control, while iSLK-BAC16-mCherry and iSLK-BAC16-mNeonGreen served as positive controls.

**Figure 3:**
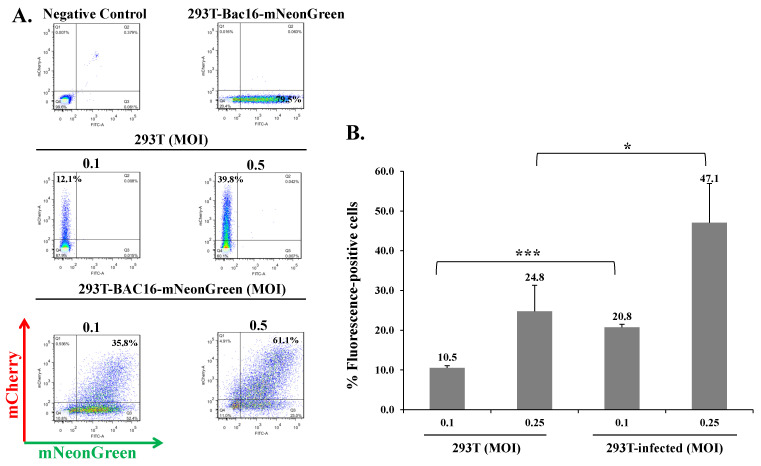
KSHV-infected HEK-293T cells can be superinfected and display enhanced expression of the fluorescent marker of the incoming virions. (**A**) Naive HEK-293T cells and latently infected HEK-293T cells that carry BAC16-mNeonGreen were seeded in six-well plates (500,000 cells per well). The next day, the cells were infected with BAC16-mCherry viruses at MOIs of 0.1 and 0.5 using spinoculation. After 48 h, the cells were harvested, and the percentages of cells expressing mNeonGreen and mCherry were measured by FACS. (**B**) Percentage of cells expressing the fluorescent protein (mNeonGreen/mCherry) encoded by the incoming viral genome. 293T—naive uninfected cells, 293T-infected-latently 293T-infected cells that were re-infected. Results shown are averages of three independent experiments. Statistical analysis was carried out using Tukey’s post hoc analysis test. *** *p* < 0.001, * *p* < 0.05. Naive uninfected HEK-293T cells were used as a negative control, while BAC16-mCherry and iSLK-BAC16-mNeonGreen-infected HEK-293T cells served as positive controls.

To investigate whether cells that are already latently infected with KSHV can be superinfected with the same virus, we combined infections with the above two recombinant viruses, BAC16-mCherry and BAC16-mNeonGreen, which enable tracking of the infection based on the expression of their fluorescent marker genes using FACS analysis. First, we used the human iSLK epithelial cells as a model system for de novo KSHV infection. These cells are susceptible to KSHV, which establishes a tightly controlled latency upon infection, and support lytic reactivation upon combined treatment with Doxycycline (Dox) and sodium butyrate (n-But) [13,57]. We infected naive and latent BAC16-mCherry-infected iSLK cells with BAC16-mNeonGreen virions at 0.1–0.5 multiplicity of infection (MOI) using the spinoculation method, and monitored expression of the mNeonGreen fluorescent marker 48 h post infection by FACS analysis. As shown in a representative experiment (Figure 2A), the percentage of mNeonGreen expressing cells in iSLK cells increased with increasing MOI and corresponded to the fractions expected using the different MOIs. Yet, at every MOI, the percentage of cells expressing mNeonGreen was higher in cells that were already latently infected with BAC16-mCherry as compared to naive initially uninfected cells. Similar results were obtained in an inverse experiment in which expression of the fluorescent marker was compared between naive and BAC16-mNeonGreen-infected cells that were infected with BAC16-mCherry virions (Figure 2B). A significant difference in the proportion of cells expressing the incoming fluorescent viral protein between naive and latently infected cells was seen at an MOI of 0.1, 0.25, and 0.5 (Figure 2C). Similar results were obtained in HEK-293T cells that were also infected at low MOIs (Figure 3). These results indicate that latently KSHV-infected cells can be superinfected with KSHV. Furthermore, latently KSHV-infected cells appear to promote enhanced expression of the fluorescent marker gene from the incoming viruses as compared with naive cells that underwent primary infection. Of note, our experiments were carried at low MOIs, thus avoiding infection with multiple virions per cell, and allowing a reliable measurement of the infected cells by FACS. At these MOIs, most fluorescent-positive cells were likely to be infected with a single infectious unit, and the variability between the infected cells in the number of incoming virions is small. Furthermore, effects resulting from high load of incoming virions that introduce large quantities of tegument proteins, which could potentially affect cells, were avoided by using low MOIs.

**Figure 4:**
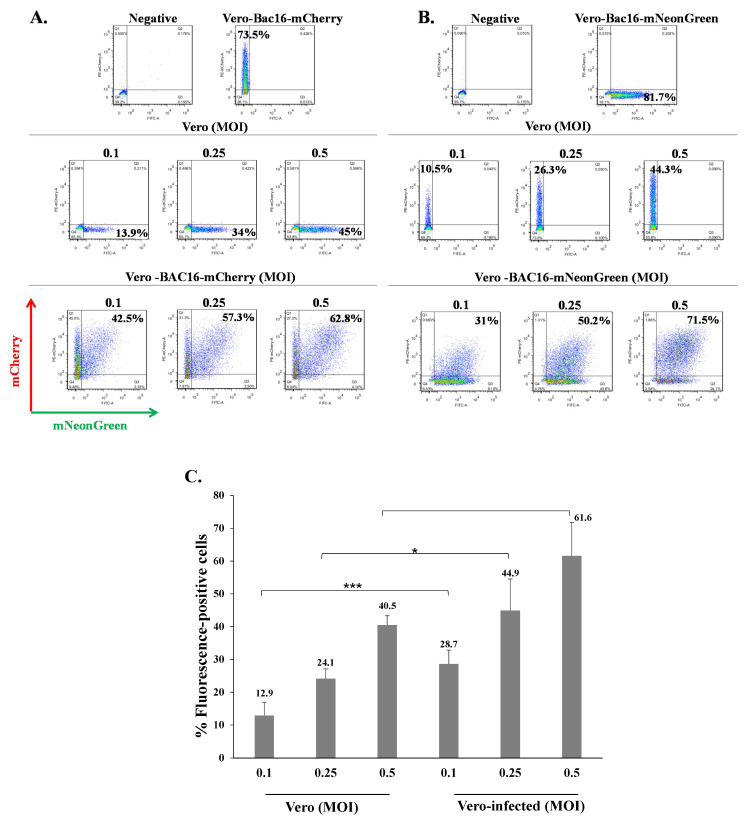
KSHV-infected Vero cells can be superinfected and display enhanced expression of the fluorescent marker of the incoming virions. (**A**) Naive Vero cells and latently infected Vero cells that carry (**A**) BAC16-mCherry or (**B**) BAC16-mNeonGreen were infected as described in Figure 2, and the percentages of cells expressing mNeonGreen and mCherry were measured by FACS. (**C**) Percentage of cells expressing the fluorescent protein (mNeonGreen/mCherry) encoded by the incoming viral genome. Vero—naive uninfected cells, Vero-infected-latently Vero-infected cells that were re-infected. Results are based on an average obtained from three independent experiments. Statistical analysis was carried out using Tukey’s post hoc analysis test. *** *p* < 0.001, * *p* < 0.05. Naive uninfected Vero cells were used as a negative control, while Vero-BAC16-mCherry and Vero-BAC16-mNeonGreen served as positive controls.

Superinfection was also evaluated in Vero cells which are incapable of interferon production, yet respond to externally added interferon [58,59]. Naive uninfected and latent BAC16-mCherry-infected Vero cells were infected with BAC16-mNeonGreen at an MOI of 0.1, 0.25, and 0.5, and the percentage of cells expressing mNeonGreen was examined 48 h later by FACS. As shown in Figure 4A, like iSLK and HEK-293T cells, the percentages of cells expressing the mNeonGreen were higher in Vero cells that were already latently infected with BAC16-mCherry as compared to naive initially uninfected cells. Similar results were obtained when BAC16-mNeonGreen-infected Vero cells were infected with BAC16-mCherry (Figure 4B), and the differences between naive and infected cells in the percentages of cells expressing the fluorescent marker from the incoming virus were significant (Figure 4C). These results indicate that Vero cells that carry latent KSHV infection are also susceptible to further KSHV infection and more efficiently express the fluorescent marker gene encoded in the genome of the incoming viruses, compared to naive cells undergoing primary infection with KSHV. This indicates that the potentially weakened interferon response in latently infected cells does not appear to be involved in the greater proportion of cells expressing the viral marker gene in latently infected cells that undergo subsequent infection with KSHV.

**Figure 5:**
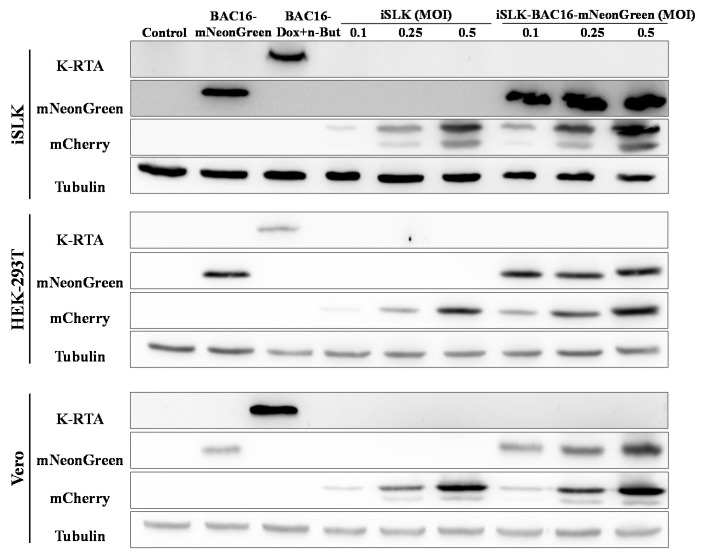
Enhanced expression of the fluorescent marker of the incoming virions in lysates from superinfected cells. Naive uninfected and latently BAC16-mNeongreen-infected iSLK, HEK-293T, and Vero cells were infected with BAC16-mCherry viruses at the indicated MOIs, as described in Figure 2. After 48 h the cells were harvested, and protein extracts were tested by WB analysis using mCherry antibody as a marker for infection with BAC16-mCherry, mNeonGreen antibody as a marker for the latent infection, and K-RTA antibody as a marker for activation of the lytic cycle. Tubulin was used as loading control. Control- uninfected iSLK, HEK-293T and Vero cells that constitute a negative control. BAC16-mNeonGreen-infected cells that were either left untreated or treated with Doxycycline (Dox, 1 µg/mL) and n-Butyrate (n-But, 1 mM) were also used to confirm mNeonGreen and RTA expression. The image represents one of two experiments that demonstrated similar results for HEK-293T cells, and one of three experiments for iSLK and Vero cells.

Similarly, WB analysis of extracts from iSLK, HEK-293T and Vero cells demonstrated higher expression of the incoming virus mCherry marker gene in extracts from latent BAC16-mNeonGreen-infected cells that were infected with BAC16-mCherry, as compared with naive cells undergoing primary infection. No expression of the immediate-early lytic viral replication and transcription activator (RTA) was evident following primary infection or superinfection (Figure 5).

**Figure 6:**
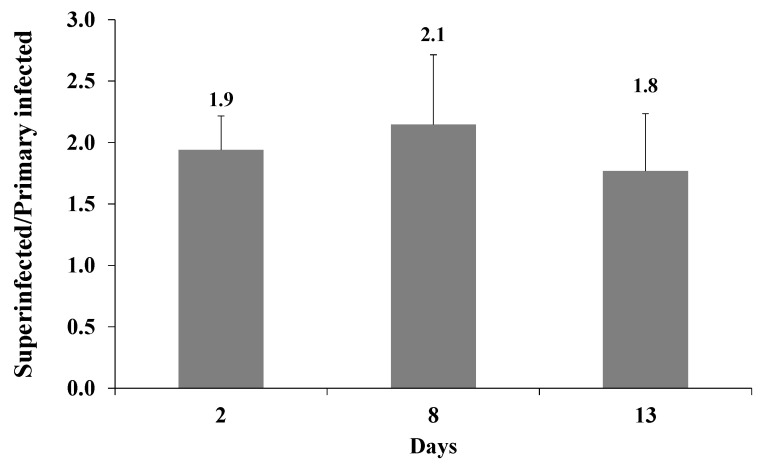
Enhanced expression of the fluorescent marker of the incoming virions in superinfected iSLK cells is maintained over time. iSLK and BAC16-mCherry or BAC16-mNeonGreen iSLK-infected cells were infected as described in Figure 2. After two, eight, and 13 d, the cells were harvested, and the percentage of cells expressing the fluorescent marker gene of the incoming virions was measured by FACS. The graph shows the ratios between the percentage of cells expressing the incoming virions fluorescent protein in superinfected and primary infected cells. Results are from three independent experiments. Statistical analysis was performed using a one-way ANOVA test, and showed no significant differences between the ratios over time.

Finally, to verify that superinfection leads to the establishment of a latent infection, we examined the expression of the incoming virion fluorescent marker protein over time, while comparing cells that were superinfected relative to primary infected iSLK cells. As shown in Figure 6, the enhanced expression of the fluorescent marker gene of the incoming virions in superinfected cells as compared to primary infected cells, which was observed 48 h post infection, was maintained at longer time points of eight and 13 d post infection. No significant differences were found in the ratios of superinfected and primary infected fluorescent cells between the different time points after infection. These results suggest that cells that are latently infected with KSHV promote establishment and maintenance of latency of additional viral genomes following superinfection.

### 2.3. Latently KSHV-Infected Cells That Were Superinfected Contain Higher Levels of the Incoming Viral DNA Compared to Primary Infected Cells

The increased fraction of cells expressing the marker gene from incoming viral genomes following infection of cells that were already latently infected with KSHV (superinfection) can be due to escape from chromatin modifications that could potentially inhibit gene expression from the viral genome, increased EF1-alpha promoter activity in latently infected cells, decreased degradation of the incoming KSHV DNA, increased efficiency of latency establishment that enables maintenance of the viral episomes during cell division, or transient replication of the viral genome upon infection. It is also possible that more than one mechanism accounts for our observation.

**Figure 7:**
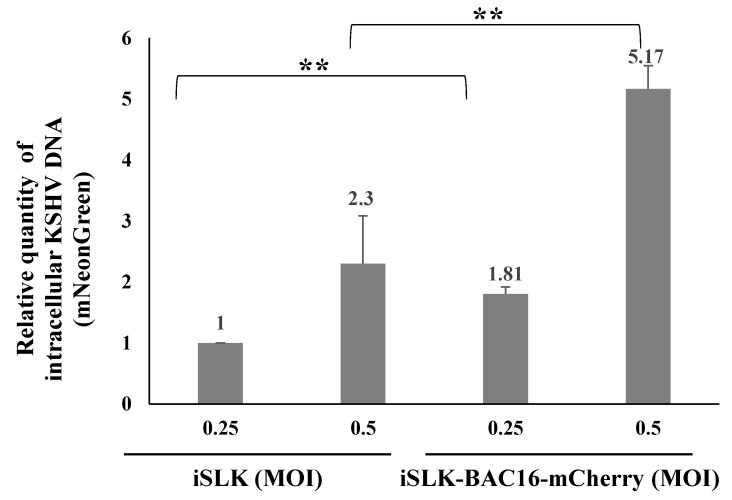
Superinfected cells contain higher levels of incoming viral DNA compared to primary infected cells. Uninfected and latently BAC16-mCherry-infected iSLK cells were infected with BAC16-mNeonGreen at MOI of 0.25 and 0.5 using the spinoculation method. High-molecular-weight DNA was extracted 48 h after infection, and the quantity of incoming viral DNA was measured using primers targeting the mNeonGreen fluorescent gene. The reactions were performed in triplicate, and the mean values obtained for the mNeonGreen fluorescent gene were normalized against the mean values obtained in the corresponding reactions of the ERV-3 cellular gene. DNA levels are shown relative to iSLK cells that were infected with BAC16-mNeonGreen virus at an MOI of 0.25, which was defined as 1 after normalization. Results obtained are from three independent experiments. Statistical analysis was performed using the ANOVA test and *t*-test. ** *p* < 0.01.

To examine these potential mechanisms, we first measured the DNA levels of the incoming viral genomes following primary infection and superinfection of the latently BAC16-mCherry-infected iSLK cells. Cells were harvested 48 h after infection, high-molecular-weight DNA was extracted, and TaqMan Real-Time PCR with primers that target the mNeonGreen fluorescent marker gene of the incoming viral genomes was used to determine the quantity of the incoming viral DNA [60]. As shown in Figure 7, the DNA load of incoming KSHV genomes was significantly higher in superinfected cells compared with primary infected cells, indicating that the increased expression of the fluorescent marker gene in superinfected cells is not due to lower suppressive chromatin modifications or increased EF1-alpha promoter activity in these cells, but instead stems from the presence of increased levels of viral genomes in the superinfected cells. Thus, it appears that differences in chromatin modifications or in the transcriptional activity of the EF1-alpha promoter do not account for the increased expression of the viral marker gene in superinfected cells.

**Figure 8:**
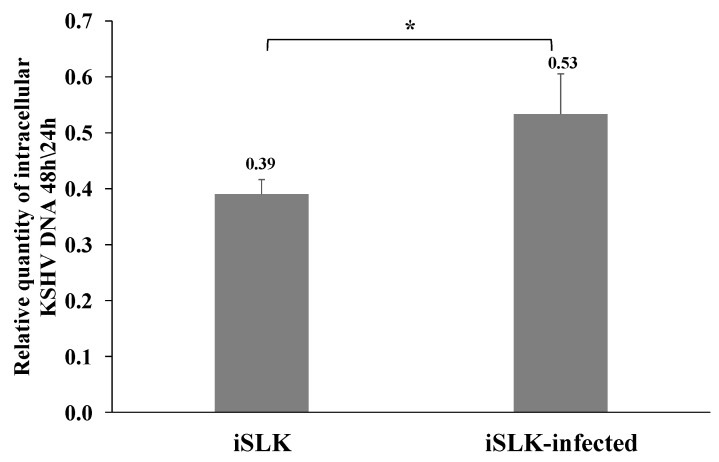
Relationship between the levels of incoming viral DNA in primary and superinfected cells 24 versus 48 h after infection. Uninfected and latently infected iSLK cells were infected with recombinant BAC16-mCherry or BAC16-mNeonGreen viruses at an MOI of 0.5 and processed as described in Figure 7 at 24 and 48 h post infection. Ratios of viral DNA levels obtained 48 and 24 h after infection are presented showing decreasing quantities of incoming viral DNA between 24 and 48 h post infection. Results obtained are from three independent experiments. Statistical analysis was performed using the Welch Two Sample *t*-test. * *p* < 0.05.

We then measured the quantity of the incoming viral DNA after superinfection and primary infection of iSLK cells 24 and 48 h post infection using primers for the fluorescent gene. Similar levels of incoming viral DNA were measured 24 h after infection in primary and superinfected cells, while the quantity of incoming viral DNA decreased at 48 h after infection. However, the reduction in the viral DNA was significantly lower in superinfected cells (ratio of 0.53 versus 0.39 between 48 and 24 h after infection in superinfected and primary infected cells, respectively) (Figure 8), suggesting smaller loss of the incoming viral DNA in already infected cells compared to cells undergoing primary infection. This finding is consistent with reduced degradation of the incoming viral DNA or higher efficiency of latency establishment prior to cell division during superinfection compared to primary infection.

**Figure 9:**
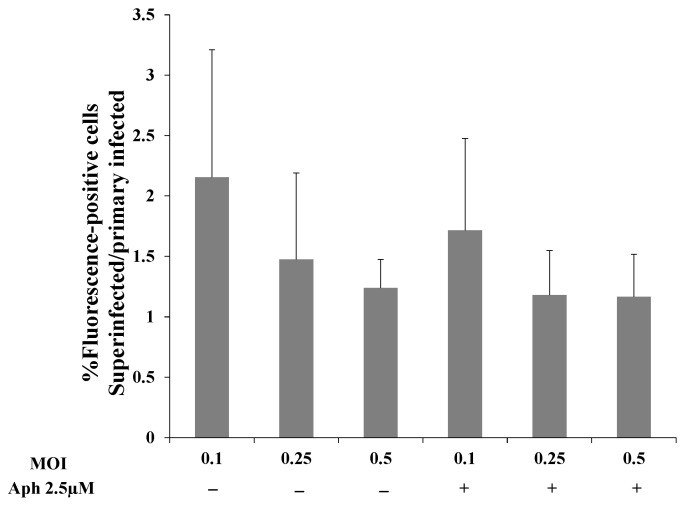
Aphidicolin treatment does not affect expression of the fluorescent marker of the incoming virions. Uninfected and latent BAC16-mCherry or BAC16-mNeonGreen-infected iSLK cells were either left untreated or treated with 2.5 µM aphidicolin (Aph) 1 h prior to infection, and infected with BAC16-mNeonGreen or BAC16-mCherry viruses at MOI of 0.1, 0.25, or 0.5 with or without aphidicolin using spinoculation. After 48 h, the cells were harvested and the percentages of cells expressing mNeonGreen and mCherry were measured by FACS. The ratios between the percentage of cells expressing the incoming virion’s fluorescent protein in superinfected and primary infected cells are presented. Results shown are averages of four independent experiments. Statistical analysis was performed using a two-way ANOVA test and differences +/− aphidicolin are not significant.

To examine whether greater replication of the incoming viral DNA or latency establishment prior to cell division account for the increased viral DNA in superinfected cells, we used aphidicolin, a potent and specific inhibitor of the B-family DNA polymerases. Aphidicolin inhibits DNA replication and cell division in eukaryotic cells and has also been reported to inhibit the DNA polymerase encoded by HSV-1 [61,62,63]. First, we determined the working concentration of aphidicolin in iSLK cells and established that 2.5 µM aphidicolin leads to cell cycle arrest without triggering cell death. Subsequently, we performed FACS experiments to examine how aphidicolin affects primary infection and superinfection. Uninfected and latently BAC16-mCherry or BAC16-mNeonGreen-infected iSLK cells were treated with aphidicolin 1 h prior to infection, or left untreated, infected with BAC16-mNeonGreen or BAC16-mCherry at an MOI of 0.1, 0.25, or 0.5 and analyzed by FACS after 48 h. In line with the results shown in Figure 2, the ratio of the fractions of cells expressing the fluorescent marker gene of the incoming virions obtained for superinfected versus primary infected cells was greater than one at all three MOIs examined. Yet, the ratios obtained for cells treated with aphidicolin versus untreated cells were not significantly different (Figure 9). These results do not support the hypothesis that increased transient replication of the incoming viral DNA in the superinfected cells relative to the primary infected cells provides an advantage to the superinfected cells. Furthermore, aphidicolin treatment is accompanied by inhibition of cell division, and therefore the advantage of superinfected cells does not appear to be due to greater preservation of the incoming viral episomes in progeny cells.

### 2.4. Increased Latency Establishment in Cells Expressing LANA-1

**Figure 10:**
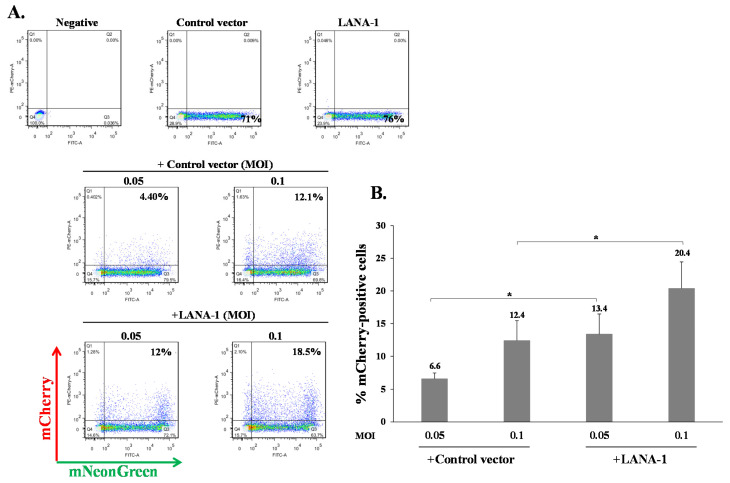
Role of LANA-1 in enhancing latency in HEK-293T. (**A**) HEK-293T cells were transfected with HA-tagged LANA-1 expression plasmid that also expressed ZsGreen fluorescent protein or with control plasmid. After 24 h, the cells were infected with BAC16-mCherry viruses at an MOI of 0.05 and 0.1 using spinoculation. Cells were harvested 48 h after infection, and the percentage of cells expressing mCherry in ZsGreen-positive cells was measured by FACS. The images represent one experiment out of three that demonstrated similar results. (**B**) Percentage of cells expressing mCherry following infection of cells transfected with LANA-1 expression vector or control vector; results shown are the average of three independent experiments. Statistical analysis was performed using the one-way ANOVA test and Tukey’s post hoc analysis tests. * *p* < 0.05.

**Figure 11:**
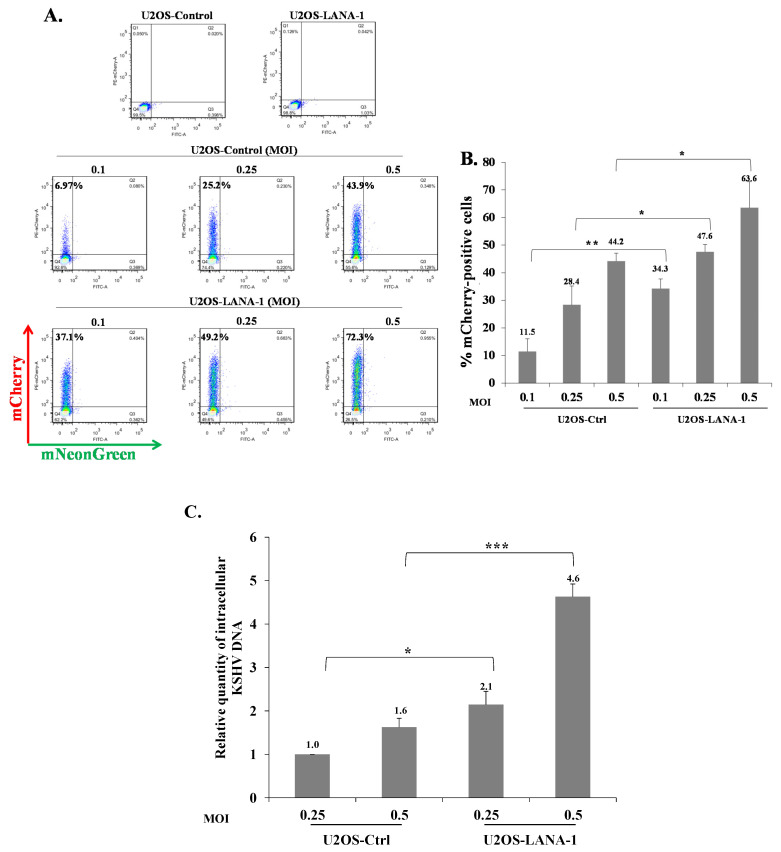
Role of LANA-1 in enhancing latency in U2OS cells. (**A**) U2OS-Control and U2OS-LANA-1 cells were treated with doxycycline (1 µg/mL), and after 24 h the cells were infected with BAC16-mCherry virus at an MOI of 0.1, 0.25, and 0.5 using spinoculation. Cells were harvested 48 h post infection and the percentages of cells expressing mCherry were measured by FACS. (**B**) Percentage of cells expressing mCherry obtained following infection based on the average of three independent experiments. Statistical analysis was performed using the One-way ANOVA test and Tukey’s post hoc analysis. * *p* < 0.05, ** *p* < 0.01. (**C**) TaqMan real-time quantitative PCR was performed as described in Figure 7. DNA levels are shown relative to U2OS-control cells that were infected with BAC16-mCherry virus at an MOI of 0.25, which was normalized to 1. Results presented are from three independent experiments. Statistical analysis was performed using the ANOVA test and *t*-test. ** *p* < 0.01, *** *p* < 0.001.

Our results indicate that KSHV-infected cells are permissive for secondary superinfection with KSHV, and that this infection results in establishment of greater latency relative to that of primary infection. This phenomenon was assumed to rely on ongoing expression of viral gene product/s that promote establishment of latency in superinfected cells. A limited number of viral gene products are expressed during latency to enable preservation and replication of the viral episomes in the infected cell nucleus, while inhibiting cell death and evading the immune response. Most of the genes expressed during latency are located in a genomic cluster known as the major latency locus, of which the latency-associated nuclear antigen 1 (LANA-1) is considered essential for the establishment and maintenance of latency in all cell types. Hence, we examined whether infection of cells that are already expressing LANA-1 results in enhanced establishment of latency. Accordingly, we transfected HEK-293T cells with an HA-tagged LANA-1 expression plasmid that also expresses ZsGreen fluorescent protein or with a control plasmid, and 24 h later, infected the cells with BAC16-mCherry viruses at an MOI of 0.05 or 0.1. mCherry expression was monitored 48 h post infection in cells expressing ZsGreen, which marked transfected cells. As shown in Figure 10A, the fraction of mCherry expressing cells in cells transfected with LANA-1 expression vector and expressing ZsGreen was higher compared to mCherry expressing cells that were transfected with the control vector. Three replicate experiments were performed, demonstrating a significantly increased mean percentage of cells expressing the marker fluorescent protein of the incoming viruses in infected cells that already expressed LANA-1 (Figure 10B). Similar results were obtained in U2OS cells that were engineered to express a doxycycline (Dox)-inducible LANA-1 protein [64]. U2OS containing LANA-1 expression cassette or control vector were treated with Dox for 24 h and then infected with BAC16-mCherry viruses at an MOI of 0.1, 0.25 and 0.5 in the presence of Dox. As shown in Figure 11A,B, cells expressing LANA-1 demonstrated higher percentages of mCherry-positive cells compared to the control cells, in which there was no LANA-1 expression. Moreover, we quantified the viral genomes in U2OS cells expressing LANA-1 compared to control cells, revealing significantly greater copy number of viral DNA in cells expressing LANA-1 compared to the control cells (Figure 11C). These results indicate that prior expression of LANA-1 in cells promotes the establishment of latent KSHV infection as evident by the relatively higher rates of fluorescent cells and greater quantities of viral DNA compared to cells that do not express this protein prior to or during infection.

### 2.5. Full-Length LANA-1 Is Required to Promote Latency Establishment of KSHV

**Figure 12:**
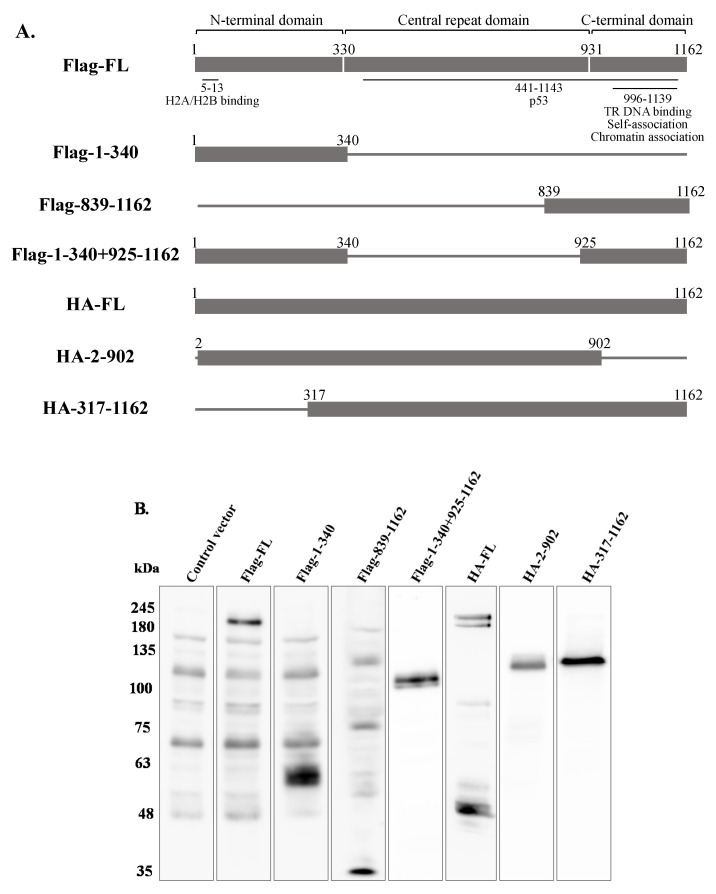

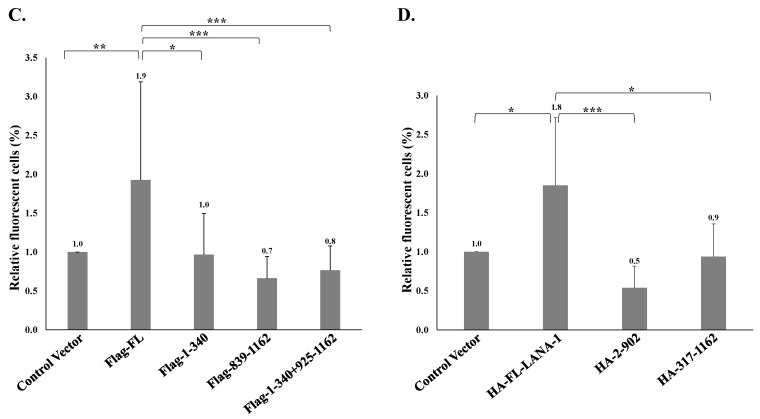
Prior expression of full-length LANA-1 is necessary to promote latency establishment. (**A**) Schematic diagram of the latency-associated nuclear antigen 1 (LANA-1) protein and fragments. HEK-293T cells were transfected with Flag or HA-tagged expression plasmids encoding different regions and full-length LANA-1. To identify the transfected cells, cells transfected with Flag-tagged expression plasmids were co-transfected with an EGFP expression plasmid, while the HA-tagged expression plasmids co-expressed ZsGreen fluorescent protein. After 24 h, the cells were infected with BAC16-mCherry viruses at an MOI of 0.25 using spinoculation. Cells were harvested 48 h after infection and the expression of LANA-1 and its corresponding fragments was analyzed by WB (**B**). The percentage of cells expressing mCherry in EGFP and ZsGreen-positive cells was measured by FACS. The value of cells transfected with a control plasmid was normalized to 1 and all samples were compared to this sample. The graph was prepared based on the average of 12 experiments for (**C**), and 8 experiments for (**D**). Statistical analysis was performed using the one-way ANOVA test and Tukey’s post hoc analysis tests. * *p* < 0.05, ** *p* < 0.01 *** *p* < 0.001.

In order to map the domain/s of LANA-1 which are necessary for promoting the establishment of latent KSHV infection in superinfected cells, we used two sets of expression plasmids encoding various fragments of this protein. To this end, we used plasmids encoding the N-terminus (aa 1–340), the C-terminus (aa 839–1162), both N- and C-termini (aa 1–340, 839–1162), and full-length LANA-1 (aa 1–1162) that were N-terminally tagged with Flag, as well as HA-tagged expression plasmids encoding the central region of the protein, which includes multiple sequence repeats, fused to the amino terminal (aa 2–902) or to the carboxy terminal (aa 317–1162) of LANA-1, and full-length LANA-1 (Figure 12A). HEK-293T cells were transfected with the different expression plasmids, and after 24 h were infected with BAC16-mCherry viruses. WB analysis confirmed the expression of LANA-1 protein and its fragments (Figure 12B) The percentage of cells expressing the fluorescent mCherry protein was measured 48 h after infection, and was significantly higher in cells transfected with the plasmids encoding Flag (Figure 12C) or HA-tagged (Figure 12D) full-length LANA-1, compared to cells transfected with control plasmids. In contrast, all tested LANA-1 fragments failed to elicit an increased percentage of cells expressing the fluorescent protein. These results indicate that expression of full-length LANA-1 protein is required to promote the establishment of latent KSHV infection in cells.

### 2.6. p53 Protein Does Not Affect Latency Establishment of KSHV during Superinfection

**Figure 13:**
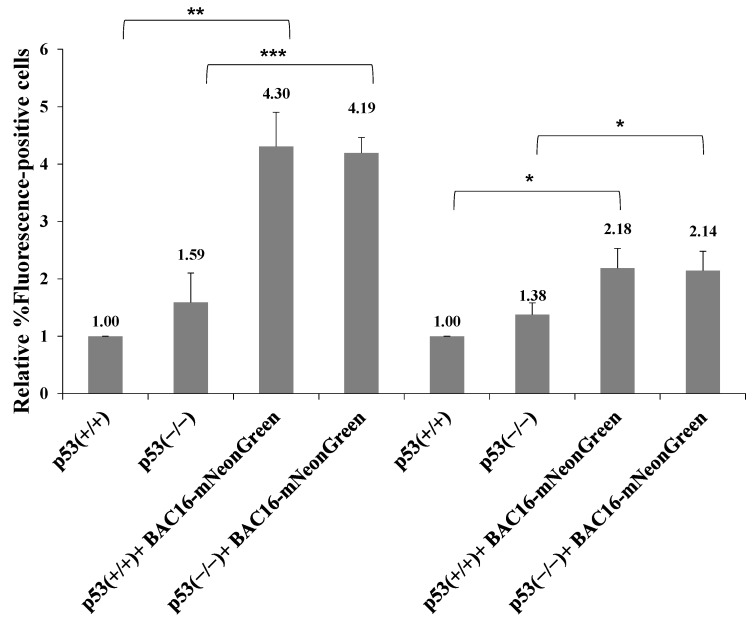
p53 protein is not involved in regulating the establishment of latent KSHV infection during superinfection. Naive HCT-116 cells expressing wild-type p53 protein (p53+/+) and an isogenic line lacking full-length p53 expression (p53−/−) as well as the corresponding BAC16-mNeonGreen-infected cells were seeded in 12-well plates (140,000 cells per well). The next day, the cells were infected with BAC16-mCherry virus at an MOI of 0.1 and 0.25 using the spinoculation method. After 48 hr, the cells were harvested, and the percentage of cells expressing mCherry was measured by FACS. Percentages obtained in HCT-p53+/+ cells were normalized to 1 and all the samples were compared to these baselines. Results shown are based on three experiments. Statistical analysis was performed using the Two-way ANOVA + Tukey’s post hoc analysis. * *p* < 0.05, ** *p* < 0.01, *** *p* < 0.001.

Previous studies showed that LANA-1 binds the p53 tumor suppressor protein and inhibits its activity while promoting cell cycle progression and induction of chromosomal instability and preserving cell survival [65,66,67]. In addition, LANA-1 participates in the ubiquitylation of p53 [68]. To examine whether p53 takes part in the LANA-1-mediated pathway promoting the establishment of latent KSHV infection, we used HCT-116 cells expressing wild-type p53 protein (HCT-p53+/+) and an isogenic line lacking full-length p53 expression (HCT-p53−/−). These cells were previously shown to enable both latency establishment and productive lytic reactivation of KSHV [69]. Both cells were infected with BAC16-mNeonGreen, and cells containing the viral episome were selected for 14 days. Primary infection and superinfection at an MOI of 0.1 and 0.25 in both cell types were characterized, as described above. As shown in Figure 13, as in iSLK, HEK-293T and Vero cells, superinfected HCT-116 cells demonstrated higher percentages of fluorescent cells. Similar efficiency of latency establishment was observed in both cell types regardless of p53 expression. Thus, in contrast to LANA-1, p53 does not appear to be involved in the promotion of latency establishment of KSHV during superinfection.

## 3. Discussion

The present study was initiated based on reports that the latency of KSHV is unstable and that cycles of lytic reactivation may be necessary to maintain long-term host infection [8,11,12]. These observations, along with reports on concomitant infection with more than one KSHV genome [47,48,49,50,51], as well as recent studies describing mutual interactions between EBV and KSHV [44,45,46], led us to examine whether cells that are latently infected with KSHV can be further re-infected with the same type of virus. Of note, in view of the prevalence of superinfection exclusion, a phenomenon whereby infection with a given virus prevents subsequent infection with the same or different type of virus, we did not know whether it would be possible to re-infect with KSHV cells that are already infected with this virus. To test the reinfection of latently infected cells, we generated new recombinant KSHV genomes expressing different fluorescent markers. The fluorescent marker genes, mCherry and mNeonGreen, do not share sequence homology nor fluorescence spectra, allowing us to conveniently track viral infections in single cells using FACS analysis. However, as gradual loss of expression of the marker gene along with retention of viral episomes has been reported in mesenchymal precursor cells [70], we also employed quantitative analysis of the incoming viral DNAs. Using several cell types, we show that infected cells that carry latent KSHV genomes can be superinfected with KSHV and that secondary infection leads to the establishment of latency of the incoming newly infecting viruses. Moreover, latency establishment of the newly penetrating viral genomes in superinfected cells was more efficient, as measured by increased proportion of fluorescent-positive cells that correlated with increased levels of the incoming viral DNA compared to primary infected cells. This phenomenon was also observed in Vero cells, which do not produce interferon alpha and beta, suggesting that the enhanced latency establishment in superinfected cells is not due to the inhibition of type I interferon response in these cells. Of note, to enable accurate tracking of infected cells while avoiding potential contribution of large quantities of viral tegument proteins, our experiments were carried at low MOIs. It is not yet known whether a similar phenomenon takes place when cells are infected at high MOI.

To further understand this phenomenon, a number of hypotheses to explain the enhanced latency establishment in superinfected cells were tested. The first hypothesis suggested a higher transcriptional activity of the EF1-alpha promoter, which controls the expression of the viral fluorescent marker genes, in superinfected cells. According to this hypothesis, the same extent of infection was obtained in primary-infected and superinfected cells, yet expression of the fluorescent marker gene of the incoming viruses was higher in the superinfected cells, and accordingly the detection of the infection in these cells increased. However, as we detected increased quantities of the incoming viral DNA (from the second infection) in superinfected cells at 48 h post infection, differences in transcriptional control cannot explain our observation. We also suggested that latency of the incoming viral genomes might be established earlier during superinfection, thereby allowing dividing cells to retain newly incoming viral genomes, whereas in primary infected cells, viral genomes could be lost during cell division. Nevertheless, the advantage of the superinfected cells remained when cells were treated with aphidicolin, which inhibits cell division. This treatment, which inhibits viral and cellular DNA polymerases, also ruled out the possibility that the incoming viral DNA replicates soon after infection and that this replication is more efficient in superinfected cells. Of note, loss of viral DNA between 24 and 48 h (Figure 8) was documented both in superinfected and primary infected cells, with primary-infected cells showing a greater decrease in the quantity of the viral DNA compared to superinfected cells. This result is consistent with the hypothesis that some of the incoming viral DNA is degraded, while in cells that are latently infected with KSHV, this process occurs at a relatively low rate resulting in a higher percentage of cells in which the fluorescent marker is expressed, higher quantities of viral DNA, and enhanced latency establishment. Of note, an increase in the quantity of incoming viral DNA, which was attributed to early lytic replication, and was followed by a decline in the viral load has been reported in PBMCs upon infection at 10 MOIs [71]. This finding is in line with our observation of reduced incoming viral DNA levels over the time frames used in the present study.

We further investigated whether ectopic expression of the major latency protein, LANA-1, prior and during infection, promotes latency establishment. Our studies revealed enhancement of latency establishment in cells expressing LANA-1, similar to that found in superinfected cells. This finding is in line with the previously reported increased stability of the viral terminal repeat (TR) plasmids transfected into cells that already express LANA-1 [8]. LANA-1 has a variety of roles including preserving viral episomes in infected cells, via parallel association with the viral origin of replication in the TR region, and histones in heterochromatin. LANA-1 has a large central repeats domain, which contributes to its episome persistence [14,72,73,74]. The predominant chromatin tethering domain of LANA-1 is found in its N-terminal region, which associates with histones H2A/H2B and various chromatin-associated proteins to facilitate association of the viral genome with metaphase chromosomes, and is essential for episome persistence [75,76,77]. The C-terminal region of LANA-1 directly binds three tandem recognition sites in the TRs that constitute a minimal origin of DNA replication [78,79], as well as host chromosome [80]. The binding of LANA-1 to the TR DNA and to host chromatin through its N- and C- terminal regions is required for latency establishment and episome maintenance, and may also influence chromatin organization of the viral episome [81,82]. The oligomerization interface of LANA-1, which was found to be important for its cooperative DNA binding to the TR, for LANA-1 nuclear body formation, and for viral genome integrity, was mapped to the C-terminus region, as well [83,84]. Of note, imaging studies revealed large nuclear multigenome clusters of KSHV episomes connected through aggregates of LANA-1 oligomers that engage binding of the N- and C- terminal regions to host chromatin and to the viral DNA [11,84,85]. The clustering of the KSHV genomes is advantageous for the replication and segregation of KSHV during latency. Thus, it is possible that latency establishment is enhanced in superinfected cells as nuclear bodies containing LANA-1 already exist in these cells, enabling recruitment of newly incoming viral DNA into pre-existing clusters. However, as ectopic expression of LANA-1 also enhances latency establishment, it is possible that the expression of sufficient quantities of LANA-1 protein functions as a limiting factor of latency establishment, while this protein is already expressed during superinfection.

Finally, as LANA-1 was shown to bind and block p53-mediated transcriptional activity, which in turn inhibits p53-induced cell apoptosis [65,66,67], we examined the involvement of p53 in the enhancement of latency establishment in superinfected cells using HCT-116 cells that either express or lack wild-type p53. However, as both cells demonstrated enhanced latency establishment during superinfection, inhibition of p53 does not appear to be involved in the enhancement of latency establishment of KSHV during superinfection.

Several cell culture models, in which the default outcome of KSHV infection is latency establishment, were used in the present study including SLK, HEK-293T, Vero, U2OS and HCT-116. All tested cell models could be re-infected with KSHV, and demonstrated enhanced latency establishment during superinfection or when full-length LANA-1 protein was expressed prior to and during infection compared to uninfected cells or cells that do not express LANA-1, respectively. These findings suggest that superinfection, in the context of natural host infection, may function to boost latently KSHV-infected cells with additional viral genomes to sustain long-term host infection with KSHV. This may also enable recombination between different KSHV genomes and thus may act as an evolutionary driving force. Finally, KS lesions contain spindle cells that are mostly latently infected with KSHV, while virions released from cells undergoing lytic infection, within or outside the lesions, may superinfect the already infected spindle cells. Such superinfection may be implicated to the pathogenesis of KS and function to sustain latency and to increase viral loads within infected cells. Similarly, although PELs are generally regarded as monoclonal malignancies, biclonal and oligoclonal patterns of KSHV episomes have been identified in PELs [86], suggesting that these neoplastic cells could originate following repeated re-infection events and be sustained through superinfection.

## 4. Materials and Methods

### 4.1. Cell Culture

Human epithelial kidney HEK-293T cells, U2OS cells (kindly provided by Meir Shamay, the Azrieli Faculty of Medicine), renal cell carcinoma SLK, and iSLK cells (kindly provided by Don Ganem, Howard Hughes Medical Institute, UCSF, San Francisco, CA, USA and Rolf Renne, University of Florida, Gainesville, FL, USA) [57] were grown in Dulbecco’s modified Eagle’s medium (DMEM) (Biological Industries, Beit Haemek, Israel), containing 50 IU/mL penicillin and 50 µg/mL streptomycin (Biological Industries, Israel), supplemented with 10% heat-inactivated fetal calf serum (FCS) (Biological Industries, Israel). U2OS cells were grown in the presence of 200 µg/mL G418 and 2 µg/mL Puromycin (A.G. Scientific Inc., San Diego, CA, USA) to maintain the Tet-on transactivator and LANA-1 expression cassette, respectively. iSLK cells were grown in the presence of 250 µg/mL G418 and 1 µg/mL Puromycin to maintain the Tet-on transactivator and the RTA expression cassette, respectively. The growth medium of BAC16-infected HEK-293T and iSLK cells was supplemented with 200 and 600 µg/mL hygromycin (MegaPharm, San Diego, CA, USA), respectively, to maintain KSHV episomes. Vero cells were grown in MEM-Eagle medium. The growth medium of BAC16-infected Vero cells was supplemented with 300 µg/mL hygromycin. HCT-116 colorectal carcinoma cells expressing wild-type p53 (p53+/+) or lacking the two p53 alleles (p53−/−) (kindly supplied by Prof. Moshe Oren, Weizmann Institute, Israel) were grown in McCoy’s 5A medium supplemented with 10% FCS [87]. Infected HCT-116 cells were selected with 500 μg/mL Hygromycin (Sigma, Kanagawa, Japan).

### 4.2. Construction of BAC16-mNeonGreen and BAC16-mCherry Recombinant Viruses

Recombinant full-length KSHV genome, cloned into a bacterial artificial chromosome (BAC16), that constitutively expresses GFP under the control of the cellular elongation factor 1-alpha (EF-1α), has been previously described, and was obtained as a kind gift from Prof. Jae Jung [55]. Construction of the recombinant viruses encoding mCherry fluorescent protein (mCherry) and mNeonGreen fluorescent protein (mNeonGreen) was accomplished using *Escherichia coli* GS1783 bacteria with a two-step recombination protocol, as described previously [56,88]. To construct a virus expressing the mCherry protein under the control of EF-1α promoter (BAC16-mCherry), we employed a previously described mCherry-transfer plasmid [89]. This plasmid was used as a PCR template to obtain a linear DNA fragment encompassing the kanamycin resistance cassette and an I-SceI restriction site, flanked by mCherry containing a small internal duplication, and the desired recombination site at positions 98,541 and 97,821 in BAC16 (GeneBank accession number GQ994935.1). The primer set used to amplify this fragment was 5′-AGCTGGCTAGGTA AGCTTGGTACCGAGCTCGGATCCACTAGTCCGCCACCATGGTGAGCAAGGGCGAGGA-3′ and 5′-TGGACGAGCTGTACAAGTAAAGCGGCCGCGACTCGGCCGCACTAGAGGAATTCCGCCCCTCTCCCTCCCC-3′ (EF1-α promoter sequence and its adjacent bases; the sequences homologous to that of the 5′ and 3′ ends of mCherry gene are underlined). To construct the recombinant KSHV BAC16 clone encoding mNeonGreen fluorescent protein under the control of EF-1α promoter (BAC16-mNeonGreen), we first generated a mNeonGreen transfer vector through the insertion of an I-SceI restriction site, a kanamycin resistance (Kan^r^) cassette, and a small internal duplication within an NcoI site in the pNCS-mNeonGreen plasmid (Allele Biotechnology, San Diego, CA, USA). This plasmid was used as a PCR template to obtain a linear DNA fragment flanked by the desired upstream and downstream recombination sites. The primer set used to amplify this fragment was: 5′-TACCGAGCTCGGATCCACTAGTCCGCCACCATGGTGAGCAAGGGCGAGGA-3′ and 5′-GCATGGACGAGCTGTACAAGTAAAGCGGCCGCGACTCGGCCGCACTAGAG-3′ (EF1-α promoter sequence and its adjacent bases: the sequences homologous to that of the 5′ and 3′ ends of mNeonGreen gene are underlined). The purified PCR products were subsequently introduced into the GS1783 E. coli strain harboring BAC16 and two-step recombination events were verified by diagnostic PCR and restriction enzyme digestion. The inserted DNA fragments were sequenced to confirm the junction sequence at the insertion site.

### 4.3. Transfection of KSHV BAC16 DNA, Virus Reconstitution, Titration and Infection

For BAC16 transfection and reconstitution, HEK-293T cells were grown to ∼70% confluence in a 24-well plate, followed by transfection with 500 ng of BAC DNA using the Lipofectamine 2000 transfection reagent (Life Technologies, Invitrogen, Waltham, MA, USA). Infected cells were selected in a medium containing 200 μg/mL hygromycin B (A. G. Scientific Inc., San Diego, CA, USA). Following selection of HEK-293T cells that carry recombinant viruses, these cells were sub-cultured and mixed at a 1:1 ratio with iSLK cells. After 24 hr, a lytic virus cycle was induced by 20 ng/mL 12-*O*-tetradecanoylphorbol-13-acetate (TPA) and 1 mM sodium butyrate (Sigma). Selection medium containing 250 µg/mL G418, 1 µg/mL puromycin and 600 ng/mL hygromycin B was added 4 days later, and iSLK cells infected with the recombinant viruses were established. KSHV-infected iSLK cells were treated with 1 μg/mL doxycycline (Dox) and 1 mM sodium butyrate (Sigma), in the absence of hygromycin, puromycin, and G418, to induce K-RTA transgene expression and lytic cycle reactivation. To purify virions, 4 days after lytic induction, supernatants were collected and cleared of cells and debris by centrifugation (700× *g* for 10 min at 4 °C) and filtration (0.45 μm-pore-size cellulose acetate filters; Corning, New York, NY, USA). Virus particles were pelleted by centrifugation (40,000× *g* for 2 h at 4 °C). Infectious units were quantified based on fluorescence, as previously described [88], while infections were performed at the indicated multiplicity of infection (MOI) using spinoculation involving centrifugation at 1500× *g* at 4 °C for 60 min in the presence of 8 µg/mL polybrene. All viruses used for infection were isolated from iSLK cells following lytic reactivation, and the number of infectious units was determined in naive uninfected iSLK cells. As expected, the infection rates, as evaluated by FACS, differed between target cells, and the actual MOIs were therefore expressed as the percentages of infected cells obtained relative to the indicated naive cells.

### 4.4. Fluorescence-Activated Cell Sorting (FACS) Analysis

Cells were trypsinized, washed and suspended in 0.5 mL phosphate-buffered saline (PBS); 0.5 mL 4% formaldehyde was added, and the cells were incubated at 4 °C for 20 min. Cells were then washed with PBS, and fluorescence-positive cells were determined by FACS (BD LSRFortessa™, BD Biosciences). Dead cells were excluded based on side and forward scatter profiles. Data analysis was performed using FlowJo software.

### 4.5. Quantitative TaqMan Real-Time Polymerase Chain Reaction

Total DNA was extracted by EZ-DNA Total DNA Isolation kit (Biological Industries, Israel). KSHV DNA was quantified by using a TaqMan-based real-time PCR assay with FAM-labeled fluorescent mCherry (Forward: 5′-CAGAGGCTGAAGCTGAAGG-3′; Reverse: 5′-GGAGGTGATGTCCAACTTGAT-3′; Probe: 5′-AGACCACCTACAAGGCCAAGAAGC-3′) or mNeonGreen (Forward: 5′-TTCCATCAGTACCTGCCCTA-3′; Reverse: 5′-GGAGGCACCATCTTCAAACT-3′; Probe: 5′-CCGGATACCAAGTCCATCGCACAA-3′) primers (Sigma) along with the cellular Cy5-labeled ERV3 gene primers (Forward: 5′-CATGGGAAGCAAGGGAACTAATG-3′; Reverse: 5′-CCCAGCGAGCAATACAGAAT TT-3′; Probe: 5′-TCTTCCCTCGAACCT GCACCATCAAT-3′). The total volume of the PCR reaction was 20 μL, and the reaction included 40 amplification cycles of 95 °C for 15 s and 60 °C for 45 s. Efficiency of the reaction was determined by the slope of the calibration curve. The average reaction efficiency, and the differences between mean values (Ct) of the mCherry or mNeonGreen gene were calculated. PCR reactions were run in triplicates on CFX96 Touch Real-Time PCR Detection system (Bio-Rad, Berkeley, CA, USA).

### 4.6. Antibodies and Western Blot Analysis

Cells were washed twice in cold PBS, suspended in radio-immunoprecipitation assay (RIPA) lysis buffer containing PMSF and a commercial protease inhibitor cocktail (Complete, Roche Diagnostics), and incubated on ice for 20 min. Cell debris were removed by centrifugation at 13,300× *g* for 15 min at 4 °C. Sodium-dodecyl sulfate (SDS) loading buffer was added, and the samples were boiled for 5 min. Protein lysates were resolved by SDS-PAGE and transferred to nitrocellulose membranes using Trans blot turbo RTA midi nitrocellulose transfer kit (Bio-Rad, Berkeley, CA, USA). The protein content of different samples was verified by Ponceau S staining. The nitrocellulose membranes were blocked with 5% dry milk in Tris-buffered saline (TBS), and subsequently incubated with primary rabbit anti mNeonGreen (Cell Signaling Technology, Danvers, MA, USA) or primary mouse antibodies to mCherry (Clontech), K-Rta (Ueda et al., 2002) (kindly provided by Keiji Ueda), or Tubulin (E7-S, DSHB). Immunoreactive bands were detected using anti-mouse or anti-rabbit antibodies conjugated to horseradish peroxidase (Jackson ImmunoResearch Laboratories, Inc., West Grove, PA, USA), and visualized using the EZ-enhanced chemiluminescence (ECL) detection kit (Clarity Western ECL Substrate, BioRad). 

### 4.7. Plasmids and Transfections

Full-length and fragments of LANA-1 containing N-terminal Flag tag were previously described [90,91]. Full-length and fragments of LANA-1 containing N-terminal HA were generated by PCR and cloned into NotI and NheI site within the DsRed expression plasmid, which also contains a ZsGreen fluorescent protein expression cassette. These plasmids were transfected into HEK-293T cells using the calcium-phosphate method.

## References

[1] Mesri E.A., Cesarman E., Boshoff C. (2010). Kaposi’s sarcoma and its associated herpesvirus. Nat. Rev. Cancer.

[2] Polizzotto M.N., Uldrick T.S., Hu D., Yarchoan R. (2012). Clinical Manifestations of Kaposi Sarcoma Herpesvirus Lytic Activation: Multicentric Castleman Disease (KSHV-MCD) and the KSHV Inflammatory Cytokine Syndrome. Front. Microbiol..

[3] Kalt I., Masa S.R., Sarid R. (2009). Linking the Kaposi’s sarcoma-associated herpesvirus (KSHV/HHV-8) to human malignancies. Methods Mol. Biol..

[4] Katano H. (2018). Pathological Features of Kaposi’s Sarcoma-Associated Herpesvirus Infection. Adv. Exp. Med. Biol..

[5] Chang Y., Cesarman E., Pessin M.S., Lee F., Culpepper J., Knowles D.M., Moore P.S. (1994). Identification of herpesvirus-like DNA sequences in AIDS-associated Kaposi’s sarcoma. Science.

[6] Goncalves P.H., Ziegelbauer J., Uldrick T.S., Yarchoan R. (2017). Kaposi sarcoma herpesvirus-associated cancers and related diseases. Curr. Opin. HIV AIDS.

[7] Gramolelli S., Schulz T.F. (2014). The role of Kaposi Sarcoma-associated Herpesvirus in the pathogenesis of Kaposi Sarcoma. J. Pathol..

[8] Grundhoff A., Ganem D. (2004). Inefficient establishment of KSHV latency suggests an additional role for continued lytic replication in Kaposi sarcoma pathogenesis. J. Clin. Investig..

[9] Aneja K.K., Yuan Y. (2017). Reactivation and Lytic Replication of Kaposi’s Sarcoma-Associated Herpesvirus: An Update. Front. Microbiol..

[10] Uppal T., Jha H.C., Verma S.C., Robertson E.S. (2015). Chromatinization of the KSHV Genome During the KSHV Life Cycle. Cancers.

[11] Chiu Y.F., Sugden A.U., Fox K., Hayes M., Sugden B. (2017). Kaposi’s sarcoma-associated herpesvirus stably clusters its genomes across generations to maintain itself extrachromosomally. J. Cell. Biol..

[12] Haddad C.O., Kalt I., Shovman Y., Xia L., Schlesinger Y., Sarid R., Parnas O. (2021). Targeting the Kaposi’s sarcoma-associated herpesvirus genome with the CRISPR-Cas9 platform in latently infected cells. Virol. J..

[13] Toth Z., Brulois K., Lee H.R., Izumiya Y., Tepper C., Kung H.J., Jung J.U. (2013). Biphasic euchromatin-to-heterochromatin transition on the KSHV genome following de novo infection. PLoS Pathog..

[14] Pei Y., Wong J.H., Robertson E.S. (2020). Herpesvirus Epigenetic Reprogramming and Oncogenesis. Annu. Rev. Virol..

[15] Sun R., Tan X., Wang X., Wang X., Yang L., Robertson E.S., Lan K. (2017). Epigenetic Landscape of Kaposi’s Sarcoma-Associated Herpesvirus Genome in Classic Kaposi’s Sarcoma Tissues. PLoS Pathog..

[16] Uppal T., Banerjee S., Sun Z., Verma S.C., Robertson E.S. (2014). KSHV LANA—The master regulator of KSHV latency. Viruses.

[17] De Leo A., Calderon A., Lieberman P.M. (2020). Control of Viral Latency by Episome Maintenance Proteins. Trends Microbiol..

[18] Folimonova S.Y. (2012). Superinfection exclusion is an active virus-controlled function that requires a specific viral protein. J. Virol..

[19] Cumby N., Davidson A.R., Maxwell K.L. (2012). The moron comes of age. Bacteriophage.

[20] Huang I.C., Li W., Sui J., Marasco W., Choe H., Farzan M. (2008). Influenza A virus neuraminidase limits viral superinfection. J. Virol..

[21] Doceul V., Hollinshead M., van der Linden L., Smith G.L. (2010). Repulsion of superinfecting virions: A mechanism for rapid virus spread. Science.

[22] Schaller T., Appel N., Koutsoudakis G., Kallis S., Lohmann V., Pietschmann T., Bartenschlager R. (2007). Analysis of hepatitis C virus superinfection exclusion by using novel fluorochrome gene-tagged viral genomes. J. Virol..

[23] Tscherne D.M., Evans M.J., von Hahn T., Jones C.T., Stamataki Z., McKeating J.A., Lindenbach B.D., Rice C.M. (2007). Superinfection exclusion in cells infected with hepatitis C virus. J. Virol..

[24] Zou G., Zhang B., Lim P.Y., Yuan Z., Bernard K.A., Shi P.Y. (2009). Exclusion of West Nile virus superinfection through RNA replication. J. Virol..

[25] Karpf A.R., Lenches E., Strauss E.G., Strauss J.H., Brown D.T. (1997). Superinfection exclusion of alphaviruses in three mosquito cell lines persistently infected with Sindbis virus. J. Virol..

[26] Biryukov J., Meyers C. (2018). Superinfection Exclusion between Two High-Risk Human Papillomavirus Types during a Coinfection. J. Virol..

[27] Johnson R.M., Spear P.G. (1989). Herpes simplex virus glycoprotein D mediates interference with herpes simplex virus infection. J. Virol..

[28] Campadelli-Fiume G., Qi S., Avitabile E., Foa-Tomasi L., Brandimarti R., Roizman B. (1990). Glycoprotein D of herpes simplex virus encodes a domain which precludes penetration of cells expressing the glycoprotein by superinfecting herpes simplex virus. J. Virol..

[29] Sloutskin A., Yee M.B., Kinchington P.R., Goldstein R.S. (2014). Varicella-zoster virus and herpes simplex virus 1 can infect and replicate in the same neurons whether co- or superinfected. J. Virol..

[30] Criddle A., Thornburg T., Kochetkova I., DePartee M., Taylor M.P. (2016). gD-Independent Superinfection Exclusion of Alphaherpesviruses. J. Virol..

[31] Meignier B., Norrild B., Roizman B. (1983). Colonization of murine ganglia by a superinfecting strain of herpes simplex virus. Infect. Immun..

[32] Roest R.W., Carman W.F., Maertzdorf J., Scoular A., Harvey J., Kant M., van der Meijden W., Verjans G.M., Osterhaus A. (2004). Genotypic analysis of sequential genital herpes simplex virus type 1 (HSV-1) isolates of patients with recurrent HSV-1 associated genital herpes. J. Med. Virol..

[33] Breuer J. (2010). VZV molecular epidemiology. Curr. Top. Microbiol. Immunol..

[34] Szpara M.L., Gatherer D., Ochoa A., Greenbaum B., Dolan A., Bowden R.J., Enquist L.W., Legendre M., Davison A.J. (2014). Evolution and diversity in human herpes simplex virus genomes. J. Virol..

[35] Norberg P., Depledge D.P., Kundu S., Atkinson C., Brown J., Haque T., Hussaini Y., MacMahon E., Molyneaux P., Papaevangelou V. (2015). Recombination of Globally Circulating Varicella-Zoster Virus. J. Virol..

[36] Bowden R., Sakaoka H., Donnelly P., Ward R. (2004). High recombination rate in herpes simplex virus type 1 natural populations suggests significant co-infection. Infect. Genet. Evol..

[37] Law G.A., Herr A.E., Cwick J.P., Taylor M.P. (2018). A New Approach to Assessing HSV-1 Recombination during Intercellular Spread. Viruses.

[38] Tomer E., Cohen E.M., Drayman N., Afriat A., Weitzman M.D., Zaritsky A., Kobiler O. (2019). Coalescing replication compartments provide the opportunity for recombination between coinfecting herpesviruses. FASEB J..

[39] Sallah N., Palser A.L., Watson S.J., Labo N., Asiki G., Marshall V., Newton R., Whitby D., Kellam P., Barroso I. (2018). Genome-Wide Sequence Analysis of Kaposi Sarcoma-Associated Herpesvirus Shows Diversification Driven by Recombination. J. Infect. Dis..

[40] Cesarman E., Chang Y., Moore P.S., Said J.W., Knowles D.M. (1995). Kaposi’s sarcoma-associated herpesvirus-like DNA sequences in AIDS- related body-cavity-based lymphomas. N. Engl. J. Med..

[41] Cesarman E. (2014). Gammaherpesviruses and lymphoproliferative disorders. Annu. Rev. Pathol..

[42] Calabro M.L., Sarid R. (2018). Human Herpesvirus 8 and Lymphoproliferative Disorders. Mediterr. J. Hematol. Infect. Dis..

[43] Sanchez-Ponce Y., Fuentes-Panana E.M. (2021). The Role of Coinfections in the EBV-Host Broken Equilibrium. Viruses.

[44] McHugh D., Caduff N., Barros M.H.M., Ramer P.C., Raykova A., Murer A., Landtwing V., Quast I., Styles C., Spohn M. (2017). Persistent KSHV Infection Increases EBV-Associated Tumor Formation In Vivo via Enhanced EBV Lytic Gene Expression. Cell Host Microbe.

[45] Bigi R., Landis J.T., An H., Caro-Vegas C., Raab-Traub N., Dittmer D.P. (2018). Epstein-Barr virus enhances genome maintenance of Kaposi sarcoma-associated herpesvirus. Proc. Natl. Acad. Sci. USA.

[46] Faure A., Hayes M., Sugden B. (2019). How Kaposi’s sarcoma-associated herpesvirus stably transforms peripheral B cells towards lymphomagenesis. Proc. Natl. Acad. Sci. USA.

[47] Gao S.J., Zhang Y.J., Deng J.H., Rabkin C.S., Flore O., Jenson H.B. (1999). Molecular Polymorphism of Kaposi’s Sarcoma-Associated Herpesvirus (Human Herpesvirus 8) Latent Nuclear Antigen: Evidence for a Large Repertoire of Viral Genotypes and Dual Infection with Different Viral Genotypes. J. Infect. Dis..

[48] Judde J.G., Lacoste V., Briere J., Kassa-Kelembho E., Clyti E., Couppie P., Buchrieser C., Tulliez M., Morvan J., Gessain A. (2000). Monoclonality or oligoclonality of human herpesvirus 8 terminal repeat sequences in Kaposi’s sarcoma and other diseases. J. Natl. Cancer Inst..

[49] Cornejo Castro E.M., Marshall V., Lack J., Lurain K., Immonen T., Labo N., Fisher N.C., Ramaswami R., Polizzotto M.N., Keele B.F. (2020). Dual infection and recombination of Kaposi sarcoma herpesvirus revealed by whole-genome sequence analysis of effusion samples. Virus Evol..

[50] Beyari M.M., Hodgson T.A., Cook R.D., Kondowe W., Molyneux E.M., Scully C.M., Teo C.G., Porter S.R. (2003). Multiple human herpesvirus-8 infection. J. Infect. Dis..

[51] Ariyoshi K., van der Loeff M.S., Cook P., Whitby D., Corrah T., Jaffarm S., Cham F., Sabally S., O’Donovan D., Weiss R.A. (1998). Kaposi’s sarcoma in the Gambia, West Africa is less frequent in human immunodeficiency virus type 2 than in human immunodeficiency virus type 1 infection despite a high prevalence of human herpesvirus 8. J. Hum. Virol..

[52] Sculley T.B., Apolloni A., Hurren L., Moss D.J., Cooper D.A. (1990). Coinfection with A- and B-type Epstein-Barr virus in human immunodeficiency virus-positive subjects. J. Infect. Dis..

[53] Yao Q.Y., Rowe M., Martin B., Young L.S., Rickinson A.B. (1991). The Epstein-Barr virus carrier state: Dominance of a single growth-transforming isolate in the blood and in the oropharynx of healthy virus carriers. J. Gen. Virol..

[54] Apolloni A., Sculley T.B. (1994). Detection of A-type and B-type Epstein-Barr virus in throat washings and lymphocytes. Virology.

[55] Brulois K.F., Chang H., Lee A.S., Ensser A., Wong L.Y., Toth Z., Lee S.H., Lee H.-R., Myoung J., Ganem D. (2012). Construction and manipulation of a new Kaposi’s sarcoma-associated herpesvirus bacterial artificial chromosome clone. J. Virol..

[56] Tischer B.K., von Einem J., Kaufer B., Osterrieder N. (2006). Two-step red-mediated recombination for versatile high-efficiency markerless DNA manipulation in Escherichia coli. Biotechniques.

[57] Myoung J., Ganem D. (2011). Generation of a doxycycline-inducible KSHV producer cell line of endothelial origin: Maintenance of tight latency with efficient reactivation upon induction. J. Virol. Methods.

[58] Mosca J.D., Pitha P.M. (1986). Transcriptional and posttranscriptional regulation of exogenous human beta interferon gene in simian cells defective in interferon synthesis. Mol. Cell. Biol..

[59] Desmyter J., Melnick J.L., Rawls W.E. (1968). Defectiveness of interferon production and of rubella virus interference in a line of African green monkey kidney cells (Vero). J. Virol..

[60] Yuan C.C., Miley W., Waters D. (2001). A quantification of human cells using an ERV-3 real time PCR assay. J. Virol. Methods.

[61] Baranovskiy A.G., Babayeva N.D., Suwa Y., Gu J., Pavlov Y.I., Tahirov T.H. (2014). Structural basis for inhibition of DNA replication by aphidicolin. Nucleic Acids Res..

[62] Pedrali-Noy G., Spadari S. (1980). Mechanism of inhibition of herpes simplex virus and vaccinia virus DNA polymerases by aphidicolin, a highly specific inhibitor of DNA replication in eucaryotes. J. Virol..

[63] Larsson A., Wraak M., Oberg B. (1983). Effect of aphidicolin on DNA synthesis in HSV-1 infected and uninfected Vero cells. Antivir. Res..

[64] Shamay M., Liu J., Li R., Liao G., Shen L., Greenway M., Hu S., Zhu J., Xie Z., Ambinder R.F. (2012). A protein array screen for Kaposi’s sarcoma-associated herpesvirus LANA interactors links LANA to TIP60, PP2A activity, and telomere shortening. J. Virol..

[65] Friborg J., Kong W., Hottiger M.O., Nabel G.J. (1999). p53 inhibition by the LANA protein of KSHV protects against cell death. Nature.

[66] Sun Z., Xiao B., Jha H.C., Lu J., Banerjee S., Robertson E.S. (2014). Kaposi’s sarcoma-associated herpesvirus-encoded LANA can induce chromosomal instability through targeted degradation of the mitotic checkpoint kinase Bub1. J. Virol..

[67] Wei F., Gan J., Wang C., Zhu C., Cai Q. (2016). Cell Cycle Regulatory Functions of the KSHV Oncoprotein LANA. Front. Microbiol..

[68] Cai Q.L., Knight J.S., Verma S.C., Zald P., Robertson E.S. (2006). EC5S ubiquitin complex is recruited by KSHV latent antigen LANA for degradation of the VHL and p53 tumor suppressors. PLoS Pathog..

[69] Gelgor A., Gam Ze Letova C., Yegorov Y., Kalt I., Sarid R. (2018). Nucleolar stress enhances lytic reactivation of the Kaposi’s sarcoma-associated herpesvirus. Oncotarget.

[70] Ellison T.J., Kedes D.H. (2014). Variable episomal silencing of a recombinant herpesvirus renders its encoded GFP an unreliable marker of infection in primary cells. PLoS ONE.

[71] Purushothaman P., Thakker S., Verma S.C. (2015). Transcriptome analysis of Kaposi’s sarcoma-associated herpesvirus during de novo primary infection of human B and endothelial cells. J. Virol..

[72] Vazquez Ede L., Carey V.J., Kaye K.M. (2013). Identification of Kaposi’s sarcoma-associated herpesvirus LANA regions important for episome segregation, replication, and persistence. J. Virol..

[73] Juillard F., Tan M., Li S., Kaye K.M. (2016). Kaposi’s Sarcoma Herpesvirus Genome Persistence. Front. Microbiol..

[74] Verma S.C., Lan K., Robertson E. (2007). Structure and function of latency-associated nuclear antigen. Curr. Top. Microbiol. Immunol..

[75] Barbera A.J., Chodaparambil J.V., Kelley-Clarke B., Joukov V., Walter J.C., Luger K., Kaye K.M. (2006). The nucleosomal surface as a docking station for Kaposi’s sarcoma herpesvirus LANA. Science.

[76] Kelley-Clarke B., De Leon-Vazquez E., Slain K., Barbera A.J., Kaye K.M. (2009). Role of Kaposi’s sarcoma-associated herpesvirus C-terminal LANA chromosome binding in episome persistence. J. Virol..

[77] Ballestas M.E., Kaye K.M. (2011). The latency-associated nuclear antigen, a multifunctional protein central to Kaposi’s sarcoma-associated herpesvirus latency. Future Microbiol..

[78] Verma S.C., Choudhuri T., Robertson E.S. (2007). The minimal replicator element of the Kaposi’s sarcoma-associated herpesvirus terminal repeat supports replication in a semiconservative and cell-cycle-dependent manner. J. Virol..

[79] Hellert J., Weidner-Glunde M., Krausze J., Lunsdorf H., Ritter C., Schulz T.F., Lührs T. (2015). The 3D structure of Kaposi sarcoma herpesvirus LANA C-terminal domain bound to DNA. Proc. Natl. Acad. Sci. USA.

[80] Matsumura S., Persson L.M., Wong L., Wilson A.C. (2010). The latency-associated nuclear antigen interacts with MeCP2 and nucleosomes through separate domains. J. Virol..

[81] Verma S.C., Choudhuri T., Kaul R., Robertson E.S. (2006). Latency-associated nuclear antigen (LANA) of Kaposi’s sarcoma-associated herpesvirus interacts with origin recognition complexes at the LANA binding sequence within the terminal repeats. J. Virol..

[82] Ottinger M., Christalla T., Nathan K., Brinkmann M.M., Viejo-Borbolla A., Schulz T.F. (2006). Kaposi’s sarcoma-associated herpesvirus LANA-1 interacts with the short variant of BRD4 and releases cells from a BRD4- and BRD2/RING3-induced G1 cell cycle arrest. J. Virol..

[83] Domsic J.F., Chen H.S., Lu F., Marmorstein R., Lieberman P.M. (2013). Molecular basis for oligomeric-DNA binding and episome maintenance by KSHV LANA. PLoS Pathog..

[84] De Leo A., Deng Z., Vladimirova O., Chen H.S., Dheekollu J., Calderon A., Myers K.A., Hayden J., Keeney F., Kaufer B.B. (2019). LANA oligomeric architecture is essential for KSHV nuclear body formation and viral genome maintenance during latency. PLoS Pathog..

[85] Shrestha P., Sugden B. (2014). Identification of properties of the Kaposi’s sarcoma-associated herpesvirus latent origin of replication that are essential for the efficient establishment and maintenance of intact plasmids. J. Virol..

[86] Boulanger E., Duprez R., Delabesse E., Gabarre J., Macintyre E., Gessain A. (2005). Mono/oligoclonal pattern of Kaposi Sarcoma-associated herpesvirus (KSHV/HHV-8) episomes in primary effusion lymphoma cells. Int. J. Cancer.

[87] Bunz F., Dutriaux A., Lengauer C., Waldman T., Zhou S., Brown J.P., Sedivy J.M., Kinzler K.W., Vogelstein B. (1998). Requirement for p53 and p21 to sustain G2 arrest after DNA damage. Science.

[88] Gelgor A., Kalt I., Bergson S., Brulois K.F., Jung J.U., Sarid R. (2015). Viral Bcl-2 Encoded by the Kaposi’s Sarcoma-Associated Herpesvirus Is Vital for Virus Reactivation. J. Virol..

[89] Bergson S., Kalt I., Itzhak I., Brulois K.F., Jung J.U., Sarid R. (2014). Fluorescent tagging and cellular distribution of the Kaposi’s sarcoma-associated herpesvirus ORF45 tegument protein. J. Virol..

[90] Krithivas A., Young D.B., Liao G., Greene D., Hayward S.D. (2000). Human herpesvirus 8 LANA interacts with proteins of the mSin3 corepressor complex and negatively regulates Epstein-Barr virus gene expression in dually infected PEL cells. J. Virol..

[91] Fujimuro M., Hayward S.D. (2003). The latency-associated nuclear antigen of Kaposi’s sarcoma-associated herpesvirus manipulates the activity of glycogen synthase kinase-3beta. J. Virol..

